# *Lactobacillus casei* and Epidermal Growth Factor Prevent Osmotic Stress-Induced Tight Junction Disruption in Caco-2 Cell Monolayers

**DOI:** 10.3390/cells10123578

**Published:** 2021-12-18

**Authors:** Geetha Samak, Rupa Rao, Radhakrishna Rao

**Affiliations:** 1 Department of Physiology, College of Medicine, University of Tennessee Health Science Center, Memphis, TN 38163, USA; geethasamak@gmail.com (G.S.); RupaRao@yahoo.com (R.R.); 2 Veterans Affairs Medical Center, Memphis, TN 38163, USA

**Keywords:** probiotic, EGF, tight junction

## Abstract

Osmotic stress plays a crucial role in the pathogenesis of many gastrointestinal diseases. *Lactobacillus casei* and epidermal growth factor (EGF) effects on the osmotic stress-induced epithelial junctional disruption and barrier dysfunction were investigated. Caco-2 cell monolayers were exposed to osmotic stress in the presence or absence of *L. casei* or EGF, and the barrier function was evaluated by measuring inulin permeability. Tight junction (TJ) and adherens junction integrity were assessed by immunofluorescence confocal microscopy. The role of signaling molecules in the *L. casei* and EGF effects was determined by using selective inhibitors. Data show that pretreatment of cell monolayers with *L. casei* or EGF attenuates osmotic stress-induced TJ and adherens junction disruption and barrier dysfunction. EGF also blocked osmotic stress-induced actin cytoskeleton remodeling. U0126 (MEK1/2 inhibitor), the MAP kinase inhibitor, blocked EGF-mediated epithelial protection from osmotic stress. In contrast, the *L. casei*-mediated epithelial protection from osmotic stress was unaffected by U0126, AG1478 (EGFR tyrosine kinase inhibitor), SP600125 (JNK1/2 inhibitor), or SB202190 (P38 MAP kinase inhibitor). On the other hand, Ro-32-0432 (PKC inhibitor) blocked the *L. casei*-mediated prevention of osmotic stress-induced TJ disruption and barrier dysfunction. The combination of EGF and *L. casei* is more potent in protecting the barrier function from osmotic stress. These findings suggest that *L. casei* and EGF ameliorate osmotic stress-induced disruption of apical junctional complexes and barrier dysfunction in the intestinal epithelium by distinct signaling mechanisms.

## 1. Introduction

Intestinal mucosal barrier function is mainly conferred by the epithelial tight junction (TJ) that prevents diffusion of allergens, toxins, and pathogens across the epithelium and subsequently into the systemic circulation [1,2]. The TJ is a multiprotein complex consisting of transmembrane proteins such as occludin, claudins, tricellulin, and junctional adhesion molecule, the intracellular scaffold proteins such as ZO-1, ZO-2, and ZO-3, and other plaque proteins such as cingulin and catenin [3,4,5,6]. The TJ protein complexes are anchored to the actin cytoskeleton, which is essential for the assembly and maintenance of TJ integrity [7,8,9]. Various signaling molecules are associated with the TJ protein complex, and intracellular signaling pathways regulate TJ integrity under physiologic and pathophysiologic conditions [10,11,12,13,14,15,16,17]. The adherens junction (AJ) that lies beneath the TJ is not a physical barrier for diffusion of macromolecules, but it indirectly regulates the integrity of TJ [9,18,19]. AJ is composed of the transmembrane protein E-cadherin, which interacts with intracellular proteins such as α-catenin, β-catenin, P120-catenin, γ-catenin, and various signaling molecules [20]. Thus, TJ and AJ in the intestine are sensitive targets for disruption by multiple types of insults, including osmotic stress.

Under normal physiological conditions, the chyme is hyperosmotic, stimulating water secretion and flow in the gut lumen during the postprandial period. The hyperosmolar stress during a meal may affect epithelial integrity [21,22]. However, mucosal protective factors in the gut may protect the epithelium from osmotic stress. If there is sustained hyperosmolarity or a compromise in the protective mechanism, it may lead to TJ disruption and barrier dysfunction. Luminal hyperosmolarity contributes to the development of colonic inflammation and mucosal damage under many pathophysiologic conditions. The hyper-osmotic fecal composition is seen in patients with Crohn’s disease, ulcerative colitis, and neonatal necrotizing enterocolitis [23,24,25,26]. Malabsorption-induced hyperosmolarity causes inflammation, diarrhea, and colitis [27,28,29,30]. Hyperosmotic stress in the gut induces pro-inflammatory cytokines and inflammatory reactions [26,31,32]. The mechanism of osmotic stress-induced mucosal damage is poorly defined. In previous in vitro and ex vivo studies, we have observed that osmotic stress disrupts the intestinal epithelial TJ and causes barrier dysfunction [32,33]. Therefore, factors that prevent osmotic stress-induced TJ disruption in the intestine likely offer therapeutic benefits in treating gastrointestinal (GI) diseases.

Epidermal growth factor (EGF), an essential mucosal protective growth factor in the GI tract, is secreted in saliva and other GI secretions [34]. EGF plays a crucial role in preventing and mitigating GI lesions [35]. Mucosal protection by other factors such as L-glutamine and probiotics is mediated by EGF receptor (EGFR) transactivation [36,37,38,39]. Probiotics are non-pharmacological agents to promote gut health and prevent or treat various GI diseases [40,41,42]. *Lactobacillus casei* (*L. casei*) is an extensively characterized and applied group of probiotics with beneficial effects in the GI tract [43]. The *L. casei* ATCC 393, one of the first identified strains of *the L. casei* group, exhibits distinct mucosal adhesiveness, the property essential for its beneficial function in the GI tract [44]. Experimental evidence indicates that the *L. casei* group of probiotics may help treat various diseases, including allergic enteropathy, obesity, cancer, and diarrhea [43]. Notably, *L. casei* ATCC 393 strain reduced tumor volume in an experimental colon cancer model [45] and alleviated enterotoxigenic *E. coli*-induced intestinal barrier dysfunction [46]. 

However, the effects of *L. casei* or EGF on osmotic stress-induced epithelial injury are unknown. Therefore, in this study, we investigated the effect of *L. casei* ATCC 393 and EGF on the osmotic stress-induced barrier dysfunction and disruption of apical junctional complexes in the intestinal epithelium.

## 2. Materials and Methods

### 2.1. Chemicals

Cell culture medium (DMEM), fetal bovine serum (FBS), antibiotics, and other related cell culture reagents and pre-cast sodium dodecyl sulfate (SDS)-Polyacrylamide gels were purchased from Invitrogen (Carlsbad, CA, USA). Mannitol, Fluorescein isothiocyanate (FITC)-inulin, leupeptin, aprotinin, bestatin, pepstatin A, phenylmethylsulfonyl fluoride (PMSF), Triton X-100, Streptavidin-agarose, SDS, protease inhibitors, EGFR tyrosine kinase-selective inhibitor AG1478 (4-(3-achloroanillino)-6,7-dimethoxyquinazoline), EGF, and vanadate were purchased from Sigma–Aldrich (St Louis, MO, USA). R0-32-0432 ((3-[(8*S*)-8-[(dimethylamino)methyl]-6,7,8,9-tetrahydropyrido[1,2-*a*]indol-10-yl]-4-(1-methyl-1*H*-indol-3-yl)-1*H*-pyrrole-2,5-dione hydrochloride), SP600125 (dibenzo[cd,g]indazol-6(2H)-one), SB202190 (4-(4-fluorophenyl)-2-(4-hydroxyphenyl)-5-(4-pyridyl)-1H-imidazole), and U0126 (1,4-diamino-2,3-dicyano-1,4-bis[2-inophenylthio]butadiene) were purchased from Calbiochem (San Diego, CA, USA).

AG1478 is a cell-permeable selective inhibitor of EGFR tyrosine kinase, commonly used as the EGF signaling blocker [47]. It also blocks the brain KV1.5 K^+^ channel in a non-protein–tyrosine–kinase-dependent manner, but it requires a 500-fold higher concentration than inhibition of EGFR kinase [48]. Ro-32-0432 is a cell-permeable PKC inhibitor, selective for conventional PKC isoforms (PKC α, βI, βII, γ) over Ca^2+^-dependent and atypical PKC isoforms [49]. SP600125 is a selective inhibitor of JNK, which inhibits phosphorylation of JNK [50]. SB202190 is a cell-permeable p38MAP kinase inhibitor, which binds to the ATP-binding site [51]. U0126 is a highly selective inhibitor of MEK1 and MEK2 (higher affinity for MEK1), the activator of ERK1/2 [52].

### 2.2. Antibodies

Mouse monoclonal anti-occludin (#33-1500) and rabbit polyclonal anti-ZO-1 (#40-2200) antibodies were purchased from ThermoFisher Scientific (Carlsbad, CA, USA). Mouse monoclonal anti-E-cadherin (#610404) and rabbit polyclonal anti-β-catenin (#610153) antibodies were purchased from BD Biosciences (San Jose, CA, USA). HRP-conjugated anti-mouse IgG (#12-349), HRP-conjugated anti-rabbit IgG (#12-348), anti-β-actin (#A5541) antibodies, AlexaFlour-488-conjugated anti-mouse IgG (#62197), Cy3-conjugated anti-rabbit IgG (#AP132C), rabbit polyclonal anti-JNK2(pT183/pY185) (SAB4300198) antibodies, and AlexaFlour-488-conjugated phalloidin (#49409) were purchased from Millipore Sigma (Eugene, OR, USA). All antibodies were affinity purified using the immunogen ligands. The specificity of most antibodies was tested by the siRNA-mediated knockdown of target proteins in the cells. The antibodies used have been extensively used in our laboratory as well as in other laboratories. The non-specific binding of fluorescently labeled secondary antibodies was tested by incubating cell monolayers with secondary antibodies without the primary antibodies.

### 2.3. Cell Culture

Caco-2_bbe1_ cells purchased from American Type Culture Collection (CRL-2102; ATCC, Rockville, MD, USA) were grown under standard cell culture conditions as described before [53]. DMEM containing 10% FBS (*v*/*v*), high glucose, L-glutamine, pyruvate, and fortified with penicillin, streptomycin, and gentamicin were used. Cells were seeded and grown as monolayers on polycarbonate membranes in transwell inserts (6.5, 12, or 24 mm diameter; Costar, Cambridge, MA, USA). We optimized the cell seeding to become confluent and differentiated with a polarized expression of sucrase by 11–13 days post-seeding. For some reason, it took 13–15 days in 12-mm transwells and 17–19 days in 24-mm transwells. The passage numbers for cells for seeding to transwells were between 24 and 36. We monitored cell monolayers for the development of barrier function by measuring TER intermittently. A similar number of cells/unit surface area was seeded to obtain a consistent level of basal TER. The basal TER in all conditions reached confluence was 500–600 Ω·cm^2^.

### 2.4. L. casei Culture

*L. casei* ATCC 393 was purchased from American Type Culture Collection (Bethesda, MD, USA). *L. casei* was grown in *Lactobacillus* Man–Rogosa–Sharp (MRS) broth (Difco, #0881) at 37 °C and under anaerobic conditions [54] for 30–36 h until the concentration reached ≅ 1.0 (λ 600 nm). Before use in experiments, bacteria were washed three times in phosphate-buffered saline (PBS).

For dead *L. casei* preparation, aliquots of bacteria were heated at 100 °C for 10 min and confirmed by culture of the treated samples. To prepare a 3KDF fraction, *L. casei*-conditioned medium was first filtered through 100-kDa cut-off membrane filters in Microcon^®^ Centrifugal Filters (Burlington, MA, USA). The 100-kDa cut-off filtrate was further filtered through 3-kDa cut-off filter (3KDF). The 3KDF samples were stored at −20 °C until use.

### 2.5. Osmotic Stress, L. casei, EGF, and Inhibitor Treatments

Cell monolayers in transwell inserts were incubated in DMEM with or without live *L. casei* (10^5^ CFU/mL; live or dead), 3KDF fraction, or EGF (30 nM) for 10 min, followed by incubation with or without 300 mOsM mannitol on the apical surface to induce osmotic stress [3,32]. In some experiments, cell monolayers were preincubated in DMEM with or without inhibitors, U0126 (10 μM), AG1478 (3 μM), R0-32-0432 (1 μM), SP600125 (1 μM), and SB202190 (5 μM) for 50 min before *L. casei*. After acclimatizing cell monolayers in DMEM for 60 min, osmotic stress was initiated by replacing the apical medium with hyperosmolar DMEM containing 300 mOsM of mannitol. EGF, *L. casei*, or 3KDF fraction was administered directly to the medium on apical and basal compartments by mixing 100X stock to the apical and basal medium 10 min before osmotic stress. The inhibitors were added 30 min before EGF or *L. casei* administration.

To test the beneficial effects of a combination of EGF and *L. casei*, we evaluated the effects of suboptimal doses of EGF (10 nM) and *L. casei* (10^4^ CFU/mL), alone or in combination, on osmotic stress-induced barrier dysfunction and TJ disruption. These doses were determined preliminary studies using different doses of EGF or *L. casei.*

### 2.6. Epithelial Barrier Function

The barrier function was evaluated by measuring transepithelial electrical resistance (TER) and unidirectional flux of FITC-inulin.

Measurement of TER: TER was measured as described previously [32] using a Millicell-ERS Electrical Resistance System (Millipore, Bedford, MA, USA). TER was calculated as Ω·cm^2^ by multiplying it with the surface area of the monolayer. The TER of the polycarbonate membrane in Transwells (approximately 30 Ω·cm^2^) was subtracted from all readings.

The unidirectional flux of inulin: Cell monolayers in transwells were incubated in the presence of FITC-inulin (0.5 mg/mL) in the basal well. At varying times during the treatments, 50 μL of the apical medium was withdrawn, and fluorescence was measured using a fluorescence plate reader (BioTEK Instruments, Winooski, VT, USA). The flux into the apical well was calculated as the percent of total fluorescence administered into the basal well per hour per cm^2^ surface area.

### 2.7. Immunofluorescence Microscopy

Immunofluorescence staining was performed as described before [17]. Briefly, cell monolayers were fixed (3% paraformaldehyde), permeabilized (0.2% Triton X100), and blocked (4% non-fat milk in TBST). Cells were incubated with primary antibodies (mouse monoclonal anti-occludin (1:200 dilution) and rabbit polyclonal anti-ZO-1 (1:400 dilution) antibodies or mouse monoclonal E-cadherin (1:200 dilution) and rabbit polyclonal anti-β-catenin antibodies (1:300 dilution). The initial incubation was followed by incubation with secondary antibodies (AlexaFluor 488-conjugated anti-mouse IgG and Cy3-conjugated anti-rabbit IgG antibodies; 1:100 dilutions) for another hour. AlexaFlour-488-conjugated phalloidin was used to stain F-actin. Fluorescence was examined under a Zeiss LSM 5 laser scanning confocal microscope, and images from x-y sections (1 μM) were collected using Zen software (Zeiss, White Plains, NY, USA). Images were stacked using the software Image J 2.1.0 (NIH, Bethesda, MD, USA) and processed by Adobe Photoshop 21.1.0 (Adobe Systems Inc., San Jose, CA, USA).

### 2.8. Immunoblot Analysis

Protein lysate from Caco-2 cells treated with osmotic stress with or without inhibitors was separated by SDS-polyacrylamide gel electrophoresis (7%) and transferred to PVDF membranes as described in a previous study [14]. Membranes were immunoblotted for JNK2(pT183/pY185) or β-actin antibodies (Invitrogen, Carlsbad, CA, USA), combined with HRP-conjugated anti-mouse IgG or anti-rabbit IgG secondary antibodies (Sigma–Aldrich, St. Louis, MO, USA). Blots were developed using the ECL chemiluminescence reagent (Pierce, Rockford, IL, USA) and quantitated by densitometry using ImageJ software. The density for each band was normalized to the density of the corresponding actin band.

### 2.9. Statistical Analysis

The observed data in the two different groups were compared using Student’s *t*-tests for grouped parametric data. Significance in all tests was set at a 95% or greater confidence level.

## 3. Results

### 3.1. L. Casei Prevents Osmotic Stress-Induced Barrier Dysfunction and TJ Disruption in Caco-2 Cell Monolayers

**Figure 1:**
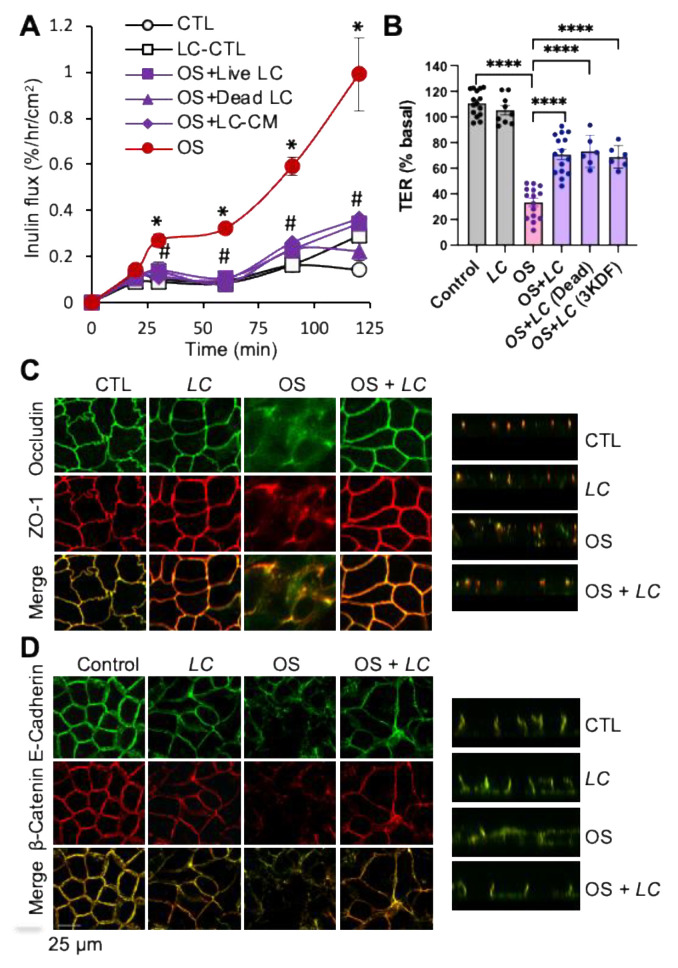
*L. casei* prevents osmotic stress-induced tight junction disruption and barrier dysfunction in Caco-2 cell monolayers. (**A**,**B**) Caco-2 cell monolayers (13–15 days post-seeding) were pretreated with (□, ■) or without (○, ●) *L. casei* (LC; 10^5^ cells/mL) for 30 min followed by osmotic stress (OS) by incubation in hyperosmotic medium (●, ■). In some groups, dead *L. casei* (▲; 10^5^ cells/mL) or 3KDF faction of *L. casei* (⧫; 5 μL/mL) instead of live *L. casei*. Inulin flux (**A**) and TER (**B**) were measured at varying times. The control (CTL) group received similar treatments in the iso-osmotic medium. Values are mean ± SEM (*n* = 3–6 from two experiments for the time course of inulin flux; *n* = 9–15 from five experiments for TER measured at 60 min after osmotic stress. In panel A, * indicates the OS values (●) that are significantly (*p* < 0.05) different from the corresponding CTL values (○); # indicates that OS + LC values (■) are significantly (*p* < 0.05) different from corresponding OS values (●). In panel B, asterisks represent significant difference between indicated groups; * = *p* < 0.05, **** = *p* < 0.0001. (**C**,**D**) At 90 min OS treatment, cell monolayers were fixed and co-stained for occludin and ZO-1 (**C**) or E-cadherin and β-catenin (**D**). X-Y and Z-section fluorescence images were captured by confocal microscopy.

To determine the epithelial protective role of *L. casei*, we evaluated its effect on osmotic stress-induced barrier dysfunction in Caco-2 cell monolayers. Osmotic stress rapidly increased the unidirectional flux of FITC-inulin (Figure 1A) and decreased TER (Figure 1B). Pretreatment of cell monolayers with *L. casei* (10^5^ CFU/mL) blocked the osmotic stress-induced increase in inulin permeability and TER decrease. These results indicate that *L. casei* prevents osmotic stress-induced epithelial barrier dysfunction.

Confocal immunofluorescence microscopy showed an osmotic stress-induced redistribution of occludin and ZO-1 from the intercellular junctions into the intracellular compartments (Figure 1C). Pretreatment with *L. casei* blocked osmotic stress-induced redistribution of TJ proteins and preserved the junctional organization of these proteins in osmotic stress-treated cell monolayers. The Z-section images confirm that *L. casei* protects the junctional organization of occludin and ZO-1 from osmotic stress-induced disruption. These results indicate that *L. casei* prevents osmotic stress-induced TJ disruption in the intestinal epithelium. Our previous study showed that osmotic stress does not induce cell death by apoptosis or necrosis in Caco-2 cells [32]. Therefore, the *L. casei* effect on barrier function is likely mediated by the prevention of TJ disruption.

To determine whether live bacteria is required for its epithelial protective effect, we evaluated the effect of dead bacteria and found that dead *L. Casei* also prevented the osmotic stress-induced decrease in TER (Figure 1B) and increase in inulin permeability (Figure 1A). Furthermore, to determine whether the protective factor from *L. casei* is released into the medium, we fractionated *L. casei* conditioned medium to prepare 3 kDa cut-off filtrate (3KDF fraction) and evaluated its effect on osmotic stress-induced barrier dysfunction. Data show that *L. casei* 3KDF fraction also prevented the osmotic stress-induced decrease in TER and increase in inulin permeability.

Since the current study involves a short-term treatment with osmotic stress, changes in the expression of tight junction proteins are not expected. Our previous study has shown that occludin and ZO-1 associated with the detergent-insoluble fraction are reduced by osmotic stress. Therefore, the short-term effect of osmotic stress is expected to reduce the redistribution of tight junction proteins. It is also expected that the proteins redistributed to cytosolic fraction are likely degraded and turned over faster than the detergent-insoluble fraction.

### 3.2. L. casei Blocks Osmotic Stress-Induced AJ Disruption

The apical junctional complex is a highly organized structure composed of TJ, AJ, and the peri-junctional actomyosin belt. We examined the effect of osmotic stress and *L. casei* on the distribution of E-cadherin and β-catenin, the AJ proteins. Confocal microscopy showed that osmotic stress caused a redistribution of E-cadherin and β-catenin from the junctions (Figure 1D). *L. casei* blocked these effects of osmotic stress of AJ proteins. The effects of osmotic stress and *L. casei* on AJ proteins were confirmed by examining the Z-section images. These data indicate that *L. casei* also prevents osmotic stress-induced AJ disruption.

### 3.3. L. casei Prevents Osmotic Stress-Induced Actin Cytoskeleton Remodeling

**Figure 2:**
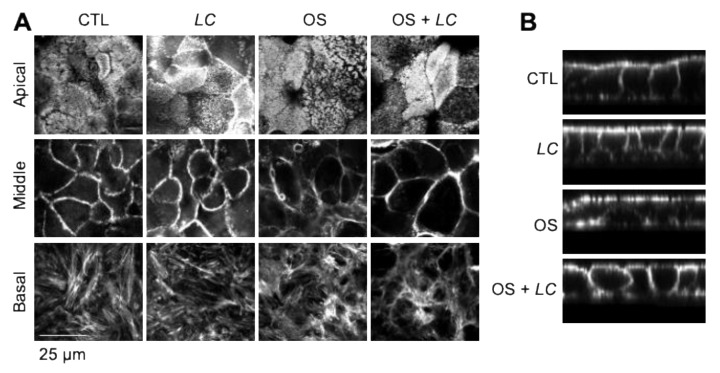
*L. casei* prevents osmotic stress-induced remodeling of the actin cytoskeleton and dissociation of junctional proteins. (**A**) Caco-2 cell monolayers (13 days post-seeding) preincubated with or without *L. casei* (LC; 10^5^ cells/mL) for 30 min, followed by osmotic stress (OS) by incubation in the hyperosmotic medium; control (CTL) monolayers received similar treatments in the iso-osmotic medium. After one hour of OS, cell monolayers were fixed and stained for F-actin using AlexaFluor-488-labeled phalloidin. Confocal fluorescence images from 2 µm optical sections at the apical, middle, and basal regions of cells are presented. (**B**) Z-section images showing the differential distribution of actin cytoskeleton at different levels of the epithelium.

Our previous studies showed that osmotic stress-induced disruption of TJ is associated with the remodeling of the actin cytoskeleton [32]. Therefore, in the current study, we examined the actin cytoskeleton organization by confocal microscopy at the epithelium’s apical, middle, and basal regions. In the apical region of control cell monolayers, actin is organized in clusters representing microvilli cross-sections. In the mid-region, actin is tightly organized in the intercellular junctions, representing the perijunctional cortical actin network, including the actomyosin belt. At the basal region, it is organized into stress fibers (Figure 2A). In osmotic stress-treated cell monolayers, the actin organization at the apical region is primarily unaffected and slightly disorganized in the basal region, but osmotic stress caused a severe disruption of actin organization in the mid-region of cells. *L. casei* treatment prevented osmotic stress-induced disruption of actin organization in the mid-region of cells. Z-section images confirmed these effects of osmotic stress and *L. casei* on the F-actin organization (Figure 2B).

### 3.4. EGF Prevents Osmotic Stress-Induced TJ Disruption and Barrier Dysfunction by a MAP Kinase-Dependent Mechanism

Previous studies have shown that EGF prevents hydrogen peroxide and acetaldehyde-induced TJ disruption and barrier dysfunction in Caco-2 cell monolayers [16,55,56,57]. Other mucosal factors such as L-glutamine and *L. plantarum* attenuated intestinal epithelial TJ disruption and barrier dysfunction by an EGFR-dependent mechanism [36,39].

**Figure 3:**
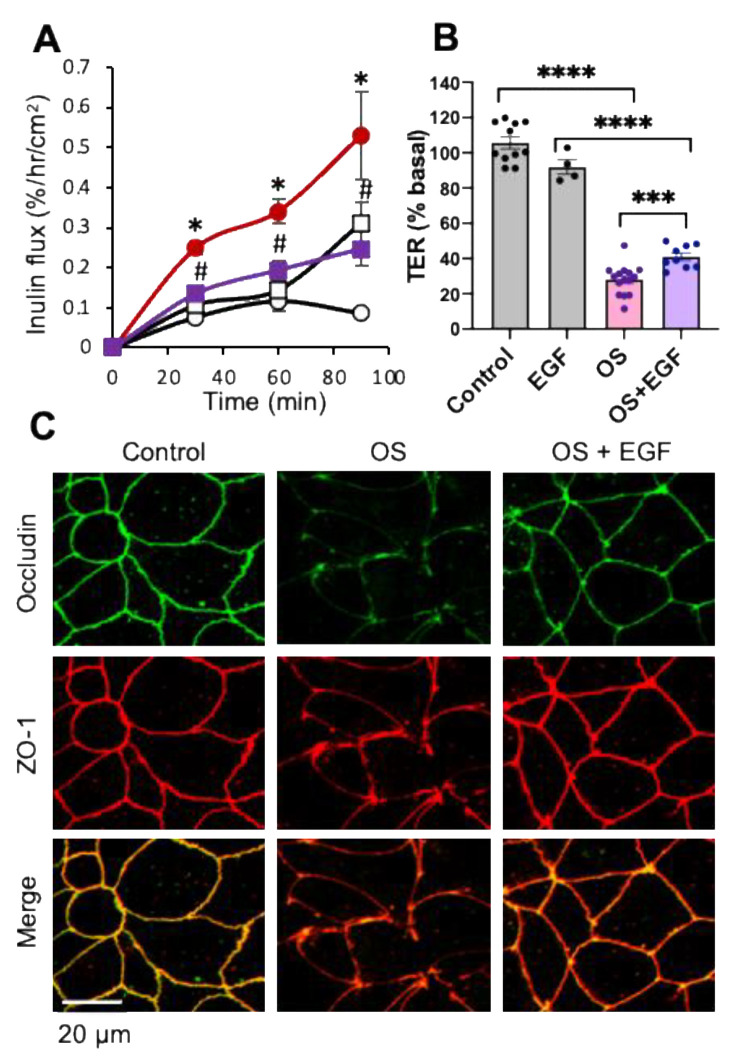
EGF prevents osmotic stress-induced tight junction disruption and barrier dysfunction in Caco-2 cell monolayers. (**A**,**B**) Caco-2 cell monolayers (13–15 days post-seeding) were pretreated with (□, ■) or without (○, ●) EGF (30 nM) for 30 min followed by osmotic stress (OS) by incubation in hyperosmotic medium (●, ■). Inulin flux (**A**) and TER (**B**) were measured at varying times. The control group received similar treatments in the iso-osmotic medium. Values are mean ± SEM (*n* = 3–17 from six experiments for the time course of inulin flux; *n* = 3–15 from five experiments for TER measured after 90 min OS). In panel A, * indicates the OS values (●) that are significantly (*p* < 0.05) different from the corresponding CTL values (○); # indicates that OS + LC values (■) are significantly (*p* < 0.05) different from corresponding OS values (●). In panel B, asterisks represent significant difference between indicated groups; *** = *p* < 0.005; **** = *p* < 0.001. (**C**) After 90 min OS treatment, cell monolayers were fixed and co-stained for occludin (green) and ZO-1 (red). Fluorescence images were captured by confocal microscopy. The orange color in merged images indicate co-localization of occludin and ZO-1.

However, the effect of EGF on the osmotic stress-induced disruption of intestinal epithelial TJ is unknown. We evaluated the effect of EGF on osmotic stress-induced TJ disruption and barrier dysfunction in the Caco-2 cell monolayers. Pretreatment of Caco-2 cell monolayers with EGF almost completely blocked the osmotic stress-induced increase in inulin permeability (Figure 3A) and partially attenuated the decrease in TER (Figure 3B). Prevention of osmotic stress-induced inulin permeability by EGF was associated with the prevention of TJ protein redistribution (Figure 3C). Data indicate that EGF prevents osmotic stress-induced TJ disruption and barrier dysfunction in the intestinal epithelium.

**Figure 4:**
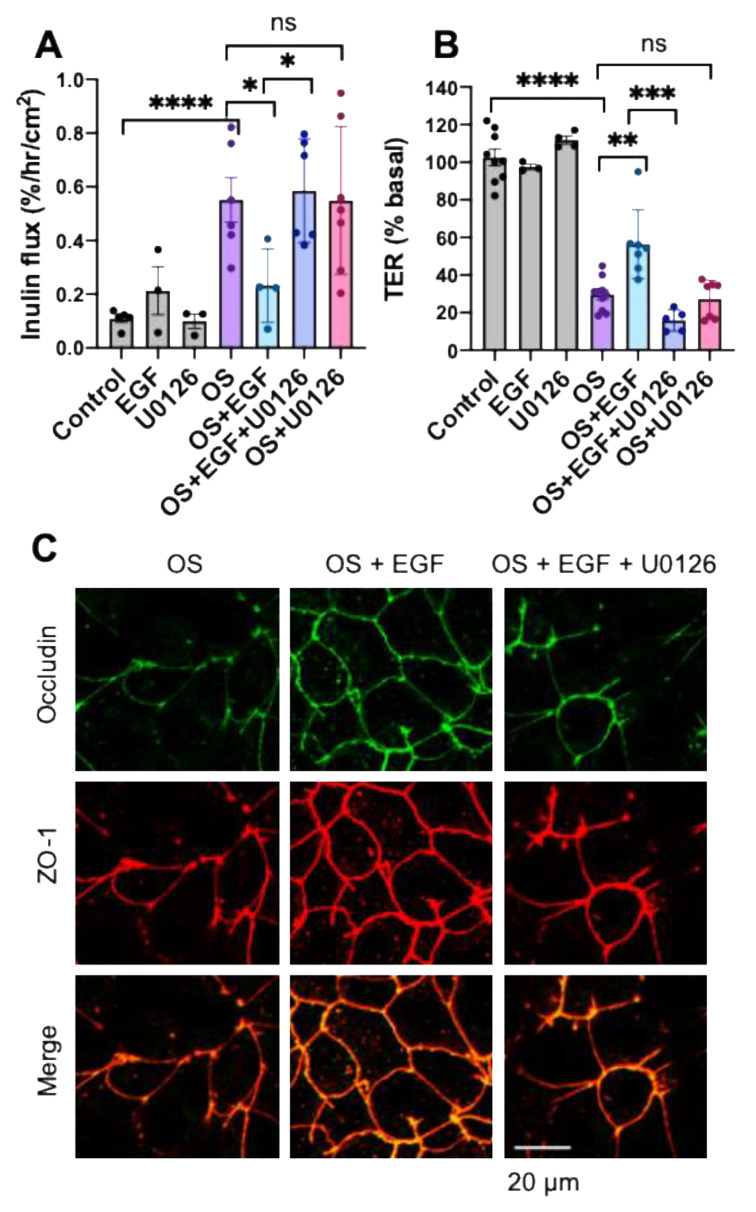
EGF prevents osmotic stress-induced tight junction disruption and barrier dysfunction by a MAP kinase-dependent mechanism. (**A**,**B**) Caco-2 cell monolayers (14 days post-seeding) were pretreated with or without U0126 (10 μM) for 30 min before incubation with EGF (30 nM). After 30 min EGF treatment, cell monolayers were exposed to osmotic stress (OS) by incubation in the hyperosmotic medium; the control group received similar treatment without EGF, U0126, or OS. Other controls included EGF-alone or U0126-alone treatments. Inulin flux (**A**) and TER (**B**) were measured at one hour of OS treatment. Values are mean ± SEM (*n* = 3-6 from two experiments for inulin flux; *n* = 3–9 from three experiments for TER); * = *p*<0.05; ** = *p* < 0.01; *** = *p* < 0.005; **** = *p* < 0.001; “ns” = not significant. (**C**) After 90 min OS treatment, cell monolayers were fixed and co-stained for occludin (green) and ZO-1 (red). Fluorescence images were captured by confocal microscopy. The orange color in merged images indicate co-localization of occludin and ZO-1.

Activation of EGFR by EGF has been shown to trigger multiple signaling pathways in the intestinal epithelium. A prominent signaling pathway in EGFR activation is the MAP kinase pathway. We evaluated the effect of U0126 (the classic inhibitor of MAP kinase pathway) on EGF-mediated protection from osmotic stress. U0126 by itself showed no effect on inulin flux or TER, but it significantly blocked EGF-mediated attenuation of osmotic stress effects on inulin permeability (Figure 4A) and TER (Figure 4B); U0126 did not alter the osmotic stress-induced changes in inulin flux or TER in the absence of EGF. U0126 also attenuated the EGF-mediated prevention of TJ protein redistribution by osmotic stress (Figure 4C).

**Figure 5:**
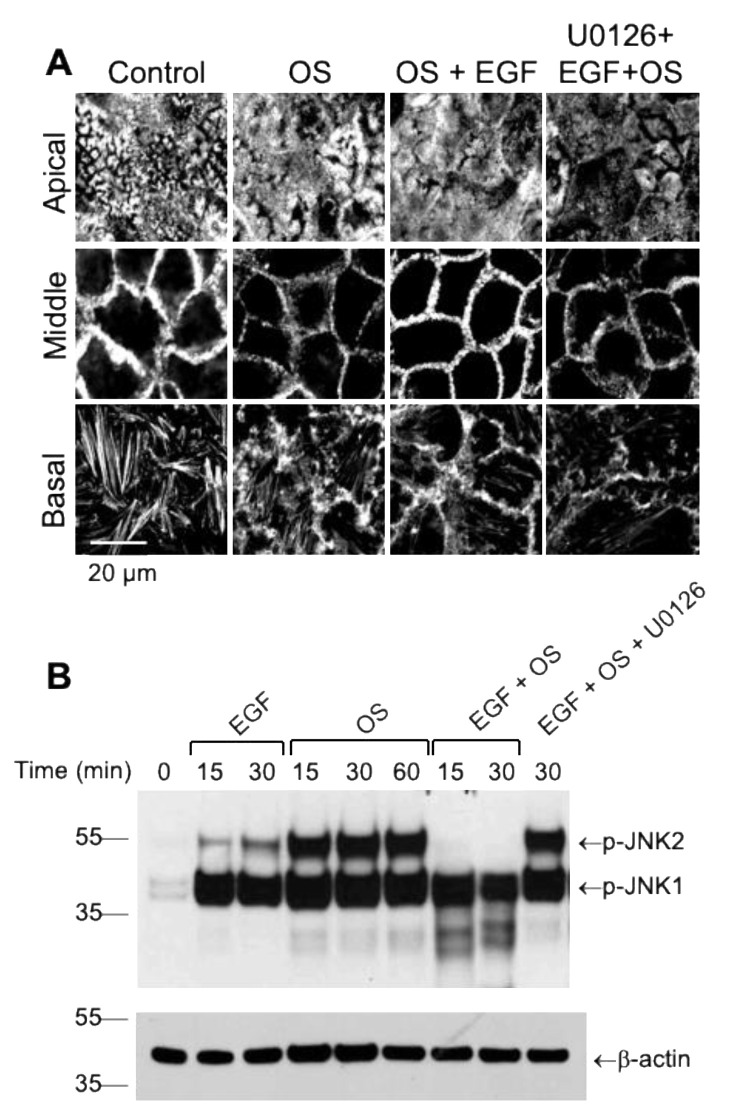
EGF prevents osmotic stress-induced actin cytoskeleton remodeling by a MAP kinase-dependent mechanism. Caco-2 cell monolayers (13 days post-seeding) were pretreated with or without U0126 (10 μM) for 30 min before incubation with EGF (30 nM). After 30 min EGF treatment, cell monolayers were exposed to osmotic stress (OS) by incubation in the hyperosmotic medium; the control group received similar treatment without EGF, U0126, or OS. (**A**) After one hour of OS treatment, cell monolayers were fixed and stained for F-actin using AlexaFluor-488-labeled phalloidin. Confocal fluorescence images from 2 µm optical sections at the apical, middle, and basal regions of the cells are presented. (**B**) Caco-2 cell monolayers (17 days post-seeding) were treated similar to panel (**A**). At varying times after OS with or without EGF and U0126, Caco-2 cell extracts were immunoblotted for JNK(pT183/pY185) and β-actin. Numbers on the left border of the images represent the molecular weight of markers.

Previous studies have also shown that osmotic stress induces remodeling of the actin cytoskeleton in Caco-2 cell monolayers. The data in the current study confirms that osmotic stress disrupts the actin cytoskeleton, particularly in the mid-region of epithelial cells. EGF treatment attenuated osmotic stress-induced actin cytoskeleton remodeling, and U0126 blocked this protective effect of EGF (Figure 5A). These data indicate that EGF stabilizes the actin cytoskeleton by a MAP kinase signaling-dependent mechanism.

Previous studies have demonstrated that osmotic stress induces rapid activation of c-Jun-N-terminal kinase-2 (JNK2), also known as a stress-activated kinase. JNK2 activity plays an essential role in osmotic stress-induced TJ disruption and barrier dysfunction in Caco-2 cell monolayers [3]. JNK activation was assessed by immunoblot analysis of cell extracts for JNK(pT183/pY185). Osmotic stress-induced a rapid and robust increase in two JNK(pT183/pY185) bands with the approximate molecular weights of 42 and 55 kDa, which likely corresponds to activated JNK1 and JNK2, respectively. The results show that EGF abrogates osmotic stress-induced activation of JNK2 (Figure 5B). U0126 blocks this effect of EGF on JNK2 activation. Interestingly, EGF or U0126 did not affect osmotic stress-induced increase in 42 kDa JNK(pT183/pY185) band, suggesting that JNK1 activation is unaffected by EGF. Data suggest that MAP kinase-mediated prevention of JNK2 activation may play a role in EGF-mediated prevention of osmotic stress-induced TJ disruption.

### 3.5. PKC But Not EGFR Tyrosine Kinase Activity Mediates L. casei-Mediated Prevention of Osmotic Stress-Induced TJ Disruption and Barrier Dysfunction

**Figure 6:**
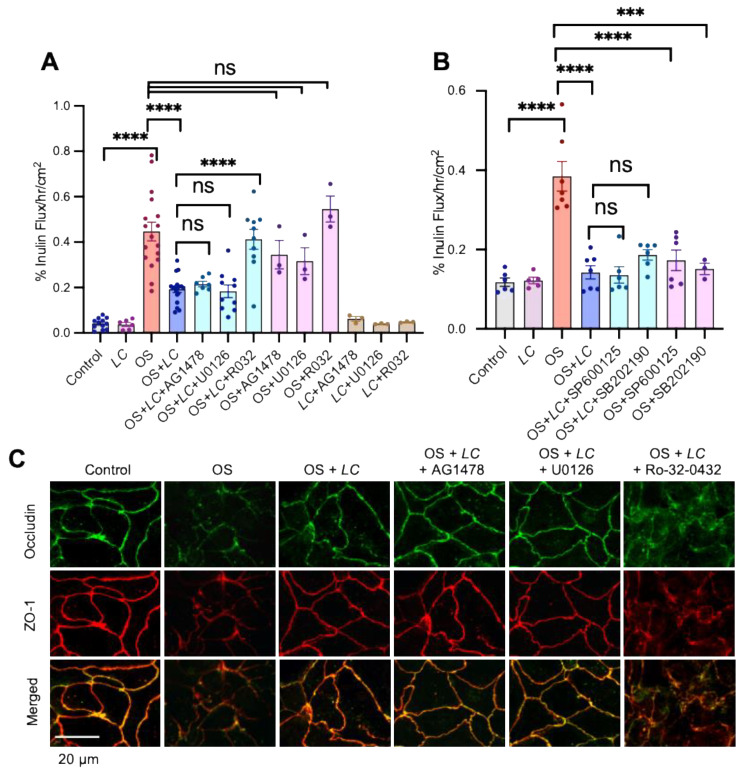
Inhibition of PKC activity blocks L. casei-mediated prevention of osmotic stress effects on the tight junction and barrier function. (**A**) Caco-2 cell monolayers (13–15 days post-seeding) were incubated with or without Ro-32-0432 (R032; the PKC inhibitor; 1 μM), AG1478 (3 μM; the EGFR tyrosine inhibitor), or U0126 (10 μM) for 30 min before *L. casei* (LC; 10^5^ cells/mL) administration. Osmotic stress (OS) was induced at 30 min LC treatment by incubation with a hyperosmotic medium. Inulin flux was measured at 90 min OS treatment. The values are mean ± SEM (*n* = 3–18 from six experiments); **** = *p* < 0.001, “ns” = not significant. (**B**) As described above, cell monolayers were treated with SP600125 (1 μM; the JNK inhibitor) or SB202190 (5 μM; the p38 MAP kinase inhibitor) before incubating with LC and OS. The inulin flux values are mean ± SEM (*n* = 3–7 from two experiments); *** = *p* < 0.005; **** = *p* < 0.001, “ns” = not significant. (**C**) After one hour of osmotic stress, cell monolayers were fixed and stained for occludin (green) and ZO-1 (red). Fluorescence images were captured by confocal microscopy. The orange or yellow color in merged images indicate co-localization of occludin and ZO-1.

Previous studies have shown that EGFR plays a crucial role in the *L. rhamnosus*-mediated protection of intestinal epithelium from hydrogen peroxide-induced TJ disruption and barrier dysfunction by a PKC-dependent mechanism [38]. In addition, prevention of alcohol-induced intestinal epithelial TJ and gut permeability by *L. plantarum* requires EGFR [39]. Therefore, we examined the potential role of EGFR and PKC activities in *L. casei*-mediated protection of TJ from osmotic stress. The *L. casei*-mediated prevention of osmotic stress effects on inulin permeability (Figure 6A) was unaffected by AG1478 (inhibitor of EGFR tyrosine kinase) or U0126. On the other hand, *L. casei*-mediated protection was blocked by Ro-32-0432 (the PKC inhibitor). AG1478, U0126, or Ro-32-0432 had no significant effect on the osmotic stress-induced increase in inulin permeability or decrease in TER. *L. casei*-mediated protection of epithelial barrier function from osmotic stress was also unaffected by SP600125 (the JNK inhibitor) or SB202190 (the P38 MAP kinase inhibitor). In contrast, these inhibitors completely blocked osmotic stress-induced inulin permeability (Figure 6B). These data indicate that the PKC activity mediates *L. casei*-mediated protection of TJ from osmotic stress but is independent of EGFR or MAP kinase activities.

Ro-32-0432, but not AG1478 or U0126, blocked *L. casei*-mediated protection from the osmotic stress-induced redistribution of occludin and ZO-1 from the intercellular junctions (Figure 6C). These results indicate that PKC activity is involved in the mechanism of *L. casei*-mediated protection of epithelial TJ from osmotic stress.

### 3.6. A Combination of EGF and L. casei Is More than Their Individual Effect in Preventing Osmotic Stress-Induced TJ Disruption and Barrier Dysfunction

**Figure 7:**
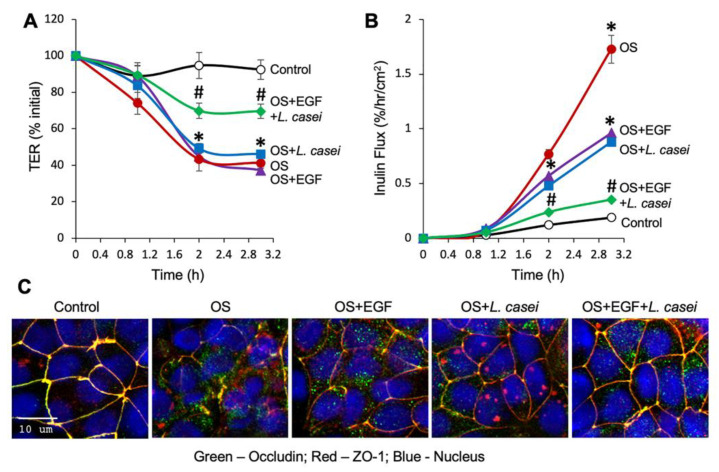
EGF and *L. casei* synergistically prevents osmotic stress-induced tight junction disruption and barrier dysfunction in Caco-2 cell monolayers. (**A**,**B**) Caco-2 cell monolayers (11-13 days post-seeding) were pretreated with 10 nM EGF (▲), *L. casei* (LC; 10^4^ cells/mL) (■), EGF + *L. casei* (⧫), or carrier (●) for 30 min followed by osmotic stress (OS) by incubation in hyperosmotic medium. The control monolayers (○) received no treatments. TER (**A**) and inulin flux (**B**) were measured at varying times. Values are mean ± SEM (*n* = 6); * indicates the values for OS with or without EGF or LC alone (●, ■, ▲) are significantly (*p* < 0.05) different from the corresponding CTL values (○); # indicates that OS+EGF+LC values (■) are significantly (*p* < 0.05) different from corresponding OS with or without EGF or LC values (●, ■, ▲). (**C**) At 3 h of OS treatment, cell monolayers were fixed and co-stained for occludin and ZO-1 and fluorescence images were captured by confocal microscopy. The orange or yellow color in merged images indicate co-localization of occludin and ZO-1.

Distinct mechanisms involved in the protective effects of EGF and *L. casei* suggested that a combination of these two epithelial protective factors may be more effective in their protective effects. Therefore, we evaluated the effect of a combination of suboptimal doses of EGF and *L. casei* on osmotic stress-induced barrier dysfunction and TJ disruption. EGF at 10-nM concentration and *L. casei* at 10^4^-CFU/mL dose showed no significant effect on osmotic stress-induced decrease in TER (Figure 7A) but showed a partial prevention of increase in inulin permeability (Figure 7B). However, the combination of EGF and *L. casei* were significantly more effective in preventing barrier dysfunction. Confocal immunofluorescence imaging of occludin and ZO-1 indicated that the combination of EGF and *L. casei* was also more effective in preventing TJ disruption (Figure 7C).

## 4. Discussion

Osmotic diarrhea is a common type of diarrhea associated with various GI diseases [58]. Disruption of epithelial tight junction is an essential passage for water to flow into the hyperosmotic lumen. Hence, blocking the osmotic disruption of TJ is an effective strategy in preventing osmotic diarrhea and leakage of toxins across the epithelium. Our current study presents evidence for the role of *L. casei* and EGF in the protection of intestinal epithelial TJ from osmotic stress. These data also suggest that distinct signaling mechanisms are involved with the epithelial protective effects of EGF and *L. casei*.

The prevention of osmotic stress-induced epithelial barrier dysfunction by *L. casei* suggests that this probiotic may offer therapeutic benefits in treating many GI diseases. The gut is the natural habitat of *L. casei*; however, its abundance in the gut is reduced in many GI diseases. Probiotic supplementation is a logical approach to correct dysbiosis and hence GI disorder. Probiotics also offer direct mucosal protective mechanisms. The protection of TJ and barrier function from various types of injury is an essential mechanism involved in the intestinal mucosal protective role of probiotics [59]. The current study demonstrates that *L. casei* prevents osmotic stress-induced disruption of TJ, AJ, and actin cytoskeleton. The actin cytoskeleton plays an essential role in the assembly and maintenance of TJ and AJ in different epithelia. Disruption of the actin cytoskeleton, such as by cytochalasin-D, leads to TJ disruption and barrier dysfunction [60]. Interestingly, osmotic stress caused severe disruption of F-actin organization in the mid-region of epithelial cells, suggesting the disruption of the actomyosin belt, where TJ and AJ interact with the actin cytoskeleton. Disruption of the actin cytoskeleton at the actomyosin belt is likely a crucial mechanism in osmotic stress-induced TJ and AJ disruption. This concept was further confirmed by osmotic stress-induced depletion of the detergent-insoluble cellular fraction of TJ proteins. The prevention of osmotic stress-induced actin remodeling in the mid-region of cells and reduction of detergent-insoluble fraction of junctional proteins by *L. casei* indicate that these effects are an integral part of the mechanism of *L. casei*-induced protection of apical junctional complexes.

Prevention of osmotic stress-induced barrier dysfunction by dead *L. casei* indicated that live *L. casei* is not required for its epithelial protective effects. Protective factor (s) expressed by these bacteria is protective to the epithelium against osmotic stress. Interestingly, the protective effect of *L. casei* 3KDF fraction indicated that the *L. casei* protective factor is released into the medium and can be isolated. This finding opens the necessity for future studies to identify this factor and characterized its structural and functional properties.

The precise mechanism of *L. casei*-mediated protection of intestinal epithelial TJ from osmotic stress is unclear. Previous studies have indicated that EGFR plays a vital role in protecting intestinal epithelial TJ and barrier function by hydrogen peroxide, ethanol, and acetaldehyde [38,39]. The current study results show that EGF protects intestinal epithelium from osmotic stress-induced TJ disruption and barrier dysfunction. The inhibitor studies indicate that the EGF-mediated epithelial protection from osmotic stress involves MAP kinase activity and actin cytoskeleton remodeling. These findings demonstrate that EGF protects the intestinal epithelial TJ and barrier function from osmotic stress by MAP kinase-mediated prevention of actin cytoskeleton remodeling. Our previous study showed that osmotic stress activates JNK2, and the JNK2 activity is involved in the mechanism of osmotic stress-induced TJ disruption and barrier dysfunction. The current study shows that EGF abolishes osmotic stress-induced activation of JNK2 by a MAP kinase-dependent mechanism. These findings demonstrate that EGF protects the intestinal epithelial TJ and barrier function from osmotic stress by MAP kinase-mediated prevention of JNK2 activation and remodeling of the actin cytoskeleton. EGF is a well-established intestinal mucosal protective factor. However, its role in the protection from osmotic damage is unknown. The current finding that EGF protects epithelial barrier function from osmotic stress suggests that EGF or agents that transactivate EGFR may have therapeutic benefits in treating various GI diseases. The mechanism of EGF-induced protection may involve activation of MAP kinase pathways leading to stabilization of TJ and actin cytoskeleton. Further studies are necessary to delineate the precise signaling pathways involved in this process.

Interestingly, AG1438 and U0126 failed to attenuate *L. casei*-mediated protection of TJ and actin cytoskeleton from osmotic stress, suggesting that EGFR tyrosine kinase or MAP kinase activities are not involved in the *L. casei* effects against osmotic damage. These results indicate that the mechanism of *L. casei* protective effect is distinctly different from the mechanism involved in the epithelial protective effects of *L. rhamnosus GG* and *L. plantarum* [38,39]. Rapid membrane translocation of PKC isoforms indicates that *L. casei* activates PKC isoforms in the intestinal epithelium. Attenuating *L. casei*-mediated prevention of osmotic stress-induced disruption of apical junctional complexes and barrier dysfunction by Ro-32-0432 suggests that PKC isoforms are involved in the mechanism of *L. casei*-mediated protection of TJ. Ro-32-0432 is known to inhibit the activities of PKCα, PKCβ1, and PKC. Our previous study showed that PKCε and PKCβI isoforms are involved in EGF-mediated remodeling of the actin cytoskeleton in Caco-2 cell monolayers [16]. The results of our current study show that *L. casei*-mediated prevention of osmotic stress-induced disruption of TJ and actin cytoskeleton requires PKC activity. Therefore, PKCβI and PKCε activities may be involved in the mechanism of *L. casei*-induced stabilization of the actin cytoskeleton, which may stabilize the TJ. A comprehensive analysis of PKC isoforms and downstream mechanisms is required for delineating the differences between mechanisms involved in EGF and *L. casei*-mediated protection of TJ. The distinct mechanisms also raise the question of whether the effects of EGF and *L. casei* on TJ are additive or synergistic. Further studies of comprehensive dose-response and time-course analyses at varying concentrations of EGF and *L. casei*, are required to address this point. The EGFR-independent mechanism involved in *L. casei* suggests that EGF and *L. casei* may have additive or synergistic effects and opens a new line investigation using multiple protective factors for effective protection against osmotic stress-induced barrier dysfunction. The findings of these studies also raise whether *L. casei* and EGF can protect the epithelial TJ and barrier function against other injurious factors such as pro-inflammatory cytokines.

The distinct intracellular mechanisms in EGF and *L. casei*-induced protection of TJ from osmotic stress raised the question of whether a combination of EGF and *L. casei* is more effective in protecting the epithelium from osmotic stress. The results of this present study show that a combination of EGF and *L casei* is more effective in preventing osmotic stress-induced TJ disruption and barrier dysfunction.

## 5. Conclusions

(1) EGF prevents osmotic stress-induced intestinal epithelial TJ disruption and barrier dysfunction by a MAP kinase-dependent mechanism. (2) *L. casei* attenuates osmotic stress-induced TJ disruption and barrier dysfunction by a PKC-dependent mechanism, but independent of EGFR and MAP kinase activities. (3) This study demonstrates that a combination of EGF and *L. casei* is more potent in preventing osmotic stress effects in the intestinal epithelium by independent signaling mechanisms and identifies the potential additive therapeutic value of these mucosal protective factors in treating GI diseases.

## Data Availability

Data from this study were submitted to FigureShare: https://figshare.com/s/68612adba63d11a79e62 (accessed on 28 January 2021). doi:10.6084/m9.figshare.13656830.
